# Effects of body mass index and extracurricular sports activities on physical fitness in school-aged children

**DOI:** 10.3389/fpubh.2025.1578304

**Published:** 2025-04-25

**Authors:** Bartosz Aniśko, Kacper Bernatowicz, Małgorzata Wójcik

**Affiliations:** ^1^Department of Physiotherapy, Faculty of Sport Sciences in Gorzów Wielkopolski, Poznań University of Physical Education, Gorzów Wielkopolski, Poland; ^2^Student Research Association Conocimiento, Faculty of Sport Sciences in Gorzów Wielkopolski, Poznań University of Physical Education, Gorzów Wielkopolski, Poland

**Keywords:** school-aged children, extracurricular sports activities, children physical fitness, children physical activity, obesity

## Abstract

**Background:**

We are seeing a steady decline in children’s physical fitness. Along with overweight and obesity, low physical fitness is one of the most serious disorders in child development. One solution to these problems is additional physical activity. It was investigated whether reduced physical fitness is associated with an increased body mass index and whether children who participate in extra-curricular sports activities have better physical fitness.

**Methods:**

The study involved 201 children (101 girls, 100 boys) aged 10 ± 2 (grades 1–8 of the primary school). Half of the participants reported participating in extra-curricular sports activities, while half did not participate in any sports activities. The children were assessed for body mass index, grip strength, balance, strength, speed and reaction time.

**Results:**

Significant differences in fitness test results were observed between active and inactive students. Significant differences were also found between students with different body mass index. The 4 months of schooling had a positive effect on most of the fitness characteristics assessed. Grip strength appeared to be a determinant of the other fitness scores.

**Conclusion:**

The hypotheses that both additional physical activity and BMI will influence children’s fitness, and that 4 months of schooling will have a positive effect on improving students’ fitness, were confirmed. Hand-grip strength was found to be a determinant of better performance in almost all other fitness tests.

## 1 Introduction

Physical fitness includes musculoskeletal and physiological features that determine the body’s ability to perform physical tasks (1, 2). Physical fitness can be defined as the capacity to perform a range of motor activities that require strength, speed, motor coordination, flexibility or endurance (3). In addition to these core components, power, reaction time and balance should also be considered (4). Physical fitness is of paramount importance, as it plays a pivotal role in a child’s optimal development. The primary determinants of fitness are physical activity, a balanced diet, and adequate sleep and rest (56). Contemporary research indicates that children and adolescents are currently failing to meet the recommended standards for physical activity, and that the prevalence of obesity is increasing due to poor dietary habits (2, 5). The physical fitness of school-aged children undergoes significant changes as they progress through different stages of development (55, 57). The educational stage of the school they are currently attending has a considerable impact on the growth of their physical fitness (59). Physical activity is known to improve both mental and physical well-being (6). Sufficient physical activity in childhood is linked to lower obesity risk, stronger muscles, better cardiovascular health, and increased bone density (7, 8). It is widely acknowledged that adequate levels of physical activity are a prerequisite for children’s physical fitness (9, 10). There is a growing body of evidence to suggest that reduced physical activity levels have a negative impact on children’s mental health, as observed during the global pandemic caused by the SARS-CoV-2 virus (11). It is crucial to ensure that children receive sufficient exercise. Extracurricular sports activities play a pivotal role in this regard, effectively complementing school physical education classes and leisure time activities. The latter should comprise a minimum of 60 min per day (12–14, 61). The available evidence indicates that most children engage in such activities on one or three occasions per week. They are most likely to participate in team games and individual sports, including swimming and cycling (60). Furthermore, it has been demonstrated that children who engage in extracurricular activities and physical activity at these activities exhibit reduced levels of fatigue (15). Physical fitness is comprised of numerous components that influence a child’s growth and performance, including strength, maximum power, static balance, speed, and reaction time. Each component has applications in everyday life. Strength generated by muscles refers to a certain power or speed over a certain period, while maximum power manifests itself in children generating as much power as possible in a short period of time. One method of measurement is the amount of maximum weight a child is able to lift (16, 17). The concept of balance can be defined as a function that necessitates the continual readjustment of muscle activity and joint position within the body to ensure the maintenance of body weight above the base of support, both in a static and dynamic state (18). The manifestation of speed is the performance of motor-related tasks and coordination in the shortest possible time, while maintaining precision. Reaction time is defined as the interval between the presentation of a stimulus and the initiation of a motor response. It is used to react as quickly as possible to external stimuli and to perform a motor activity under specific conditions in the shortest possible time (58). These components facilitate the proper functioning of children and contribute to the development of their physical fitness through training. It has been demonstrated that physical fitness, even in children aged 3–6 years, is correlated with intellectual maturity (19). It is regrettable that, in recent years, children have not met the recommended standard for time spent in physical activity. Furthermore, this time continues to decrease from adolescence through to adulthood (20–22). It has been observed that when children meet the recommended daily physical activity guidelines, this occurs primarily on days when they have physical education (PE) classes (23). This is a cause for concern, given the numerous benefits of physical activity. Despite efforts to promote physical activity and offer more sporting opportunities, many children increasingly prefer sedentary forms of entertainment, including electronic devices. A decline in the physical capabilities of children worldwide has been observed, as evidenced by reductions in strength, agility, endurance, and muscular flexibility (24). Currently, healthy lifestyles are being promoted to encourage all types of movement, healthy eating, and related activities among young people. However, it remains unclear whether this will halt the persistent decline in the percentage of children engaging in physical activity (25). There has been a notable decline in the popularity of physical education among pupils, particularly among female students (26–28). Despite the designated lesson hours for this subject, an increasing proportion of children do not participate, with this figure rising further at the next stages of education. It is evident that the large number of both parental and medical exemptions, which are often used (29), exerts a considerable influence.

## 2 Materials and methods

The objective of the study was to evaluate the physical fitness of school-aged children, with an additional assessment of the impact of physical activity, body mass index (BMI), and sex on the children under study. The study was conducted twice: the initial measurement was taken at the commencement of the academic year in October, while the subsequent measurement was conducted 4 months later in February. This allowed for an investigation of whether school-related activities would enhance the physical fitness of the children. The following hypotheses were proposed:

1. Children participating in extracurricular sports will perform significantly better in physical fitness tests than those who do not.
2. Children with higher BMI (overweight or obese) will show poorer results in most fitness tests, except for hand-grip strength.
3. Hand-grip strength will significantly correlate with results of other physical fitness tests.
4. Physical fitness test results will improve significantly after 4 months of school activities.

**Figure 1:**
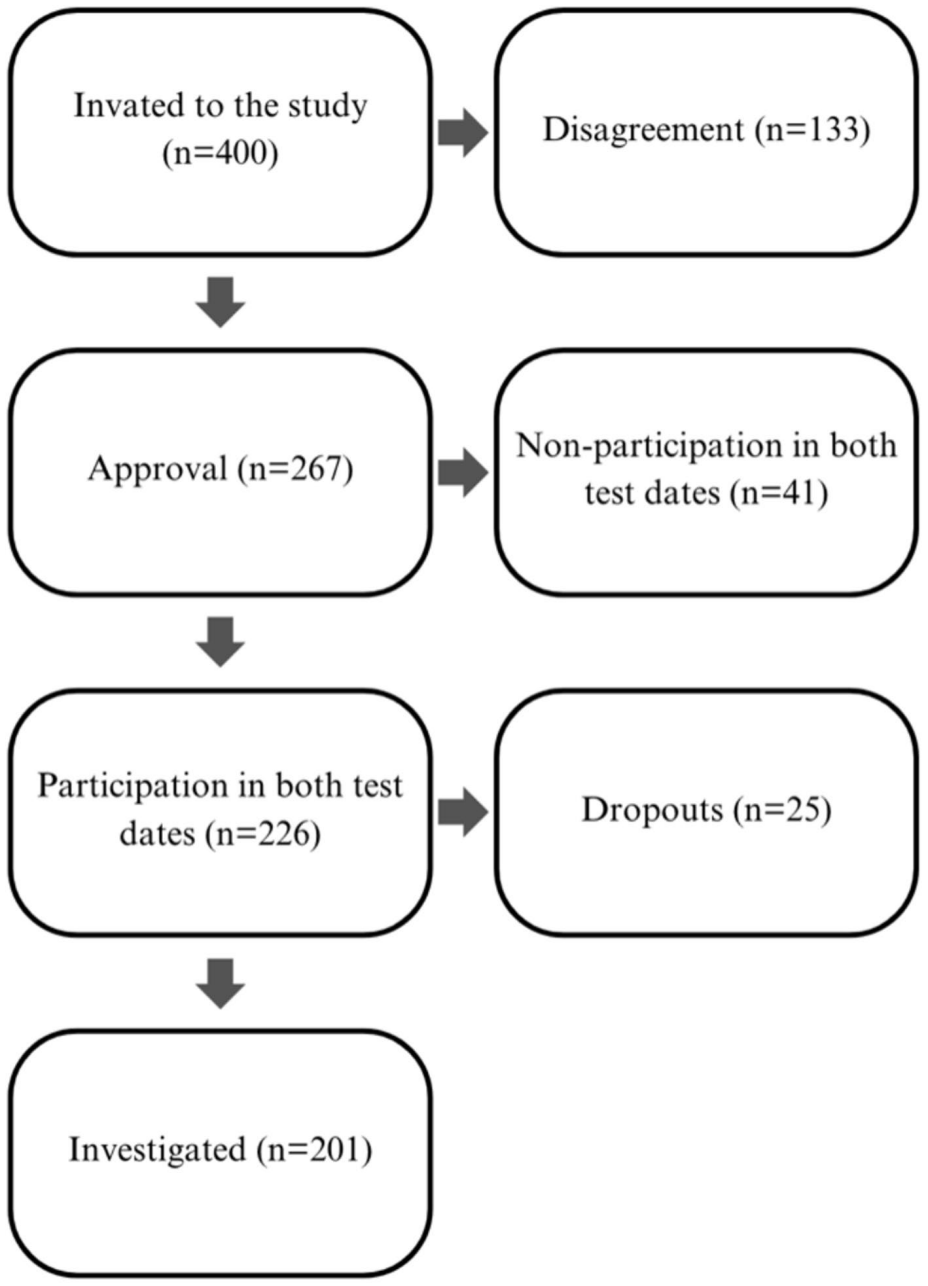
Number of study participants.

The survey was conducted with primary school students. A total of 400 individuals were invited to participate in the study, and consent was obtained from the guardians of 267 children. Ultimately, 201 individuals completed the study in its entirety (Figure 1). The study plan involved two appointments, with a three-month interval between them. To complete the study, participants were required to attend both appointments and complete all fitness tests. The study was reported to and approved by the local Bioethics Committee at the Medical University of Karol Marcinkowski in Poznań, resolution number 427/17. Only children whose parents gave written consent for their child to take part in the study were included.

### 2.1 Participants

Participants in the study were children in primary grades 1–8. Demographic data are shown in Table 1.

**Table 1:** Demographics of study participants.

Category	Number of participants (%)
Girls	101 (50.25)
Boys	100 (49.75)
City	151 (75.12)
Village	50 (24.88)

The average height of the girls studied was 145.96 ± 12.35 cm, while that of the boys was 144.84 ± 15.10 cm. The average body weight of the girls was 38.31 ± 13.04 kg, while that of the boys was 40.91 ± 16.06 kg. Participation in extra-curricular sports activities was reported by 38% of the participants, while not participating in any activities was reported by 62% (Table 2).

**Table 2:** Declaration regarding participation in extra-curricular sports activities.

Category	Percentage of participants (%)
Participation in sports activities	38
Lack of participation in sports activities	62

Among those declaring additional extra-curricular sports activities, specific activities were listed (Table 3).

**Table 3:** Type of physical activity chosen.

Type of activity	Number of participants
Football	36
Gymnastics	19
Dance	10
Horse riding	5
Combat sports	5
Rocket sports	4
Athletics	3
Running	2
Gym	1

Sixty percent of respondents were of normal weight, 12% were underweight, 21% were overweight and 6% were obese (Table 4).

**Table 4:** Body mass status according to BMI.

BMI Index	Percentage of participants (%)
Correct	60
Underweight	12
Overweight	21
Obese	6

### 2.2 Physical fitness tests

The study commenced at 8:00 AM to minimize the influence of daily physical activity on the test results. A total of eight stations were evaluated, encompassing measurements of body height, body weight, static balance, hand grip strength, lower limb power and lower limb speed.

### 2.3 Height and weight

An electronic scale altimeter (Radwag) was employed to ascertain the height and weight of the test subject. The subject was instructed to step onto the scale and assume an upright, stationary position. The researcher would then set the altimeter at the appropriate height and record the subject’s height and weight.

### 2.4 Body mass index (BMI)

To accurately interpret body mass index (BMI), centile grids created as part of the projects ‘Nationwide Study of Physical Development of Children and Adolescents’ and ‘Nationwide Anthropometric Monitoring of Children and Adolescents’, which were implemented in Poland between 2007 and 2013, were employed. A centile grid is a statistical instrument employed in the fields of medicine and research to examine the patterns of height, body weight, and other developmental indicators among children and adolescents. It is a graphical or tabular representation of the distribution of population data according to age and sex. The centile grid is a statistical tool that enables the assessment of whether an individual’s development is aligned with population norms.

### 2.5 Balance

The assessment of static balance ability was conducted using a two-plate posturograph (Koordynacja). The test subject was instructed to step onto the platform and assume a stable upright position. The feet were positioned in the center of the measuring platforms, the arms were lowered along the body, and the gaze was directed ahead. The subject was then asked to maintain a completely still position for 30 s.

### 2.6 Hand-grip strength

The grip strength of the upper limbs was evaluated using a hand dynamometer (Saehan). During the measurement, the subject was instructed to stand upright with their arms lowered along the torso, holding the dynamometer in one hand. The test subject was then required to squeeze the dynamometer with maximum force first with their right hand and then with their left hand.

### 2.7 Lower limb power and speed

Lower limb power and speed were quantified using the Optogait optical system (MicroGate Timing). The system comprises two optical sensors positioned at a distance of 1.5 m apart. The subject was required to enter the designated measurement area and await the commencement signal while wearing appropriate athletic footwear. The initial assessment was conducted to determine the maximum power of the lower limbs. The participant was instructed to perform three maximal jumps upwards within a 10-s timeframe. Subsequently, the participant was required to assess the speed of the lower limbs while remaining within the designated measuring field. This was achieved through the performance of foot tapping on the spot (i.e., picking up and touching the ground with the feet again as quickly as possible). The test commenced and concluded with the presentation of an audible signal.

### 2.8 Reaction time

The measurement of the reaction time was conducted using the Microgate Wittysem optical system. The system comprises four semaphores, namely special photocells that register the intersection of the light beam. The two outermost semaphores were positioned at a height of 140 cm, while the two central semaphores were situated at a height of 170 cm. The distance between the semaphores was 50 cm. During the test, different symbols were displayed at random, and the subject’s task was to react as quickly as possible to the designated symbol. The program used involved responding to the lowercase letter “a” in green. The entire test consisted of 20 repetitions.

### 2.9 Statistical analysis

All statistical analysis and data visualization were conducted using the Statistica 13 and R package (30). The analysis was based on a series of physical performance variables, including balance, reaction time (s), maximal power (W), lower limb movement speed (repetitions), hand-grip strength (N), body height (cm) and body weight (kg). The normality of the distribution of the analyzed data was assessed using the Shapiro–Wilk test, while the homogeneity of the variances of the groups was evaluated through the application of the Levene’s test. The data on left-hand grip strength did not satisfy the criteria for homogeneity of variance; therefore, the non-parametric Kruskal-Wallis test was employed to assess the differences between the groups. The remaining data exhibited homogeneity of variance, thus enabling the application of an ANOVA analysis. To accurately characterize the differences between specific groups, a *post hoc* analysis using Dunn’s test with Bonferroni adjustment for multiple comparisons. Was applied (*p* < 0.05: *, *p* < 0.01: **, *p* < 0.001: ***). To ascertain whether there were any statistically significant differences between the active and inactive groups, the Wilcoxon test was employed. Conversely, the Spearman coefficient was utilized to assess the strength of the association between the variables. Effect sizes were calculated using Cohen’s *d* for repeated measures and the rank-biserial correlation coefficient (*r*) for independent group comparisons, in line with the nature of the applied statistical tests. The results were then presented in tabular and graphical form.

## 3 Results

**Figure 2:**
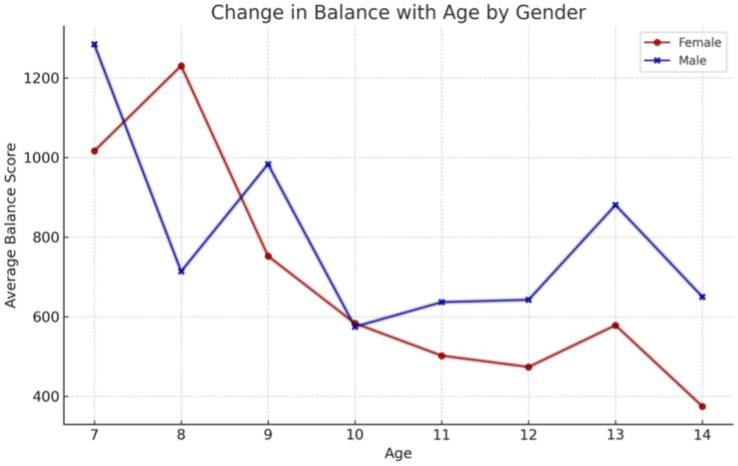
The changes observed with age were found to be in equilibrium with the sex of the subjects.

**Figure 3:**
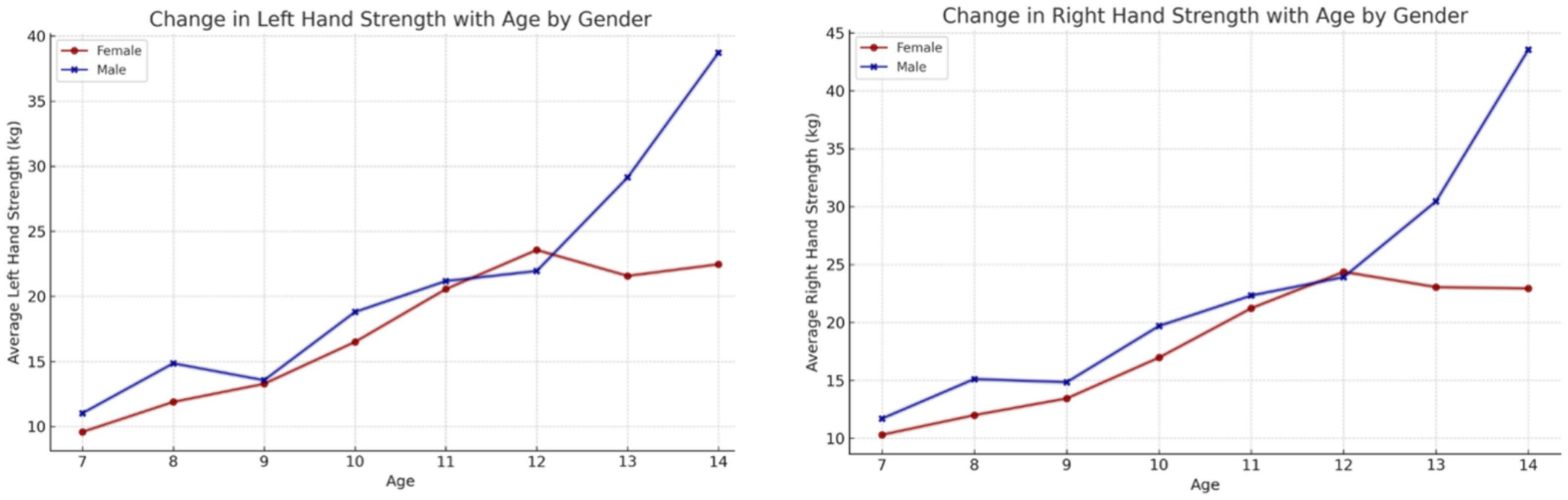
Impact of age on right and left limb compression strength, with a particular focus on sex differences.

In the initial phase of the data analysis, the impact of age and sex on physical fitness was evaluated. The findings revealed that static balance ability exhibited a gradual improvement in older children, irrespective of sex (Figure 2). A comparison of the left and right limb grip strength reveals an increase that remains at the same level in both sexes until the age of 12 years (Figure 3). Thereafter, a distinct increase in strength is observed in boys, while girls exhibit a plateau at 20–25 kg.

**Figure 4:**
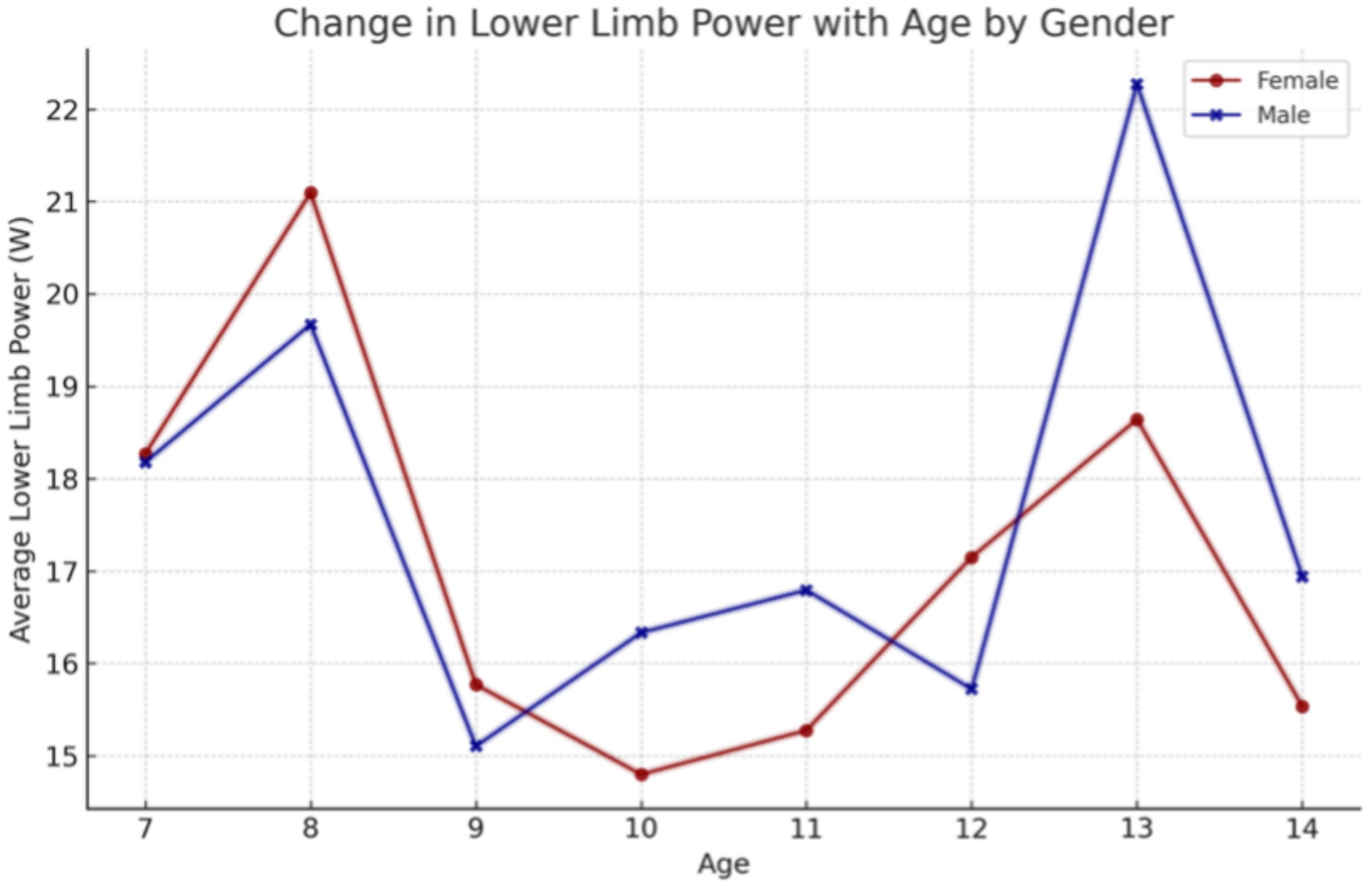
Impact of age on lower limb power, with a particular focus on the influence of sex.

The results for changes in lower limb maximal power exhibited variability according to the age of the subjects (Figure 4). Children aged 7–8 years demonstrated average results of 18 to 21 watts (W), while children aged 8–12 years exhibited average results in the 15–18 W range. After the age of 12 years, a dynamic increase was observed, particularly in boys, followed by another decrease after 13 years.

**Figure 5:**
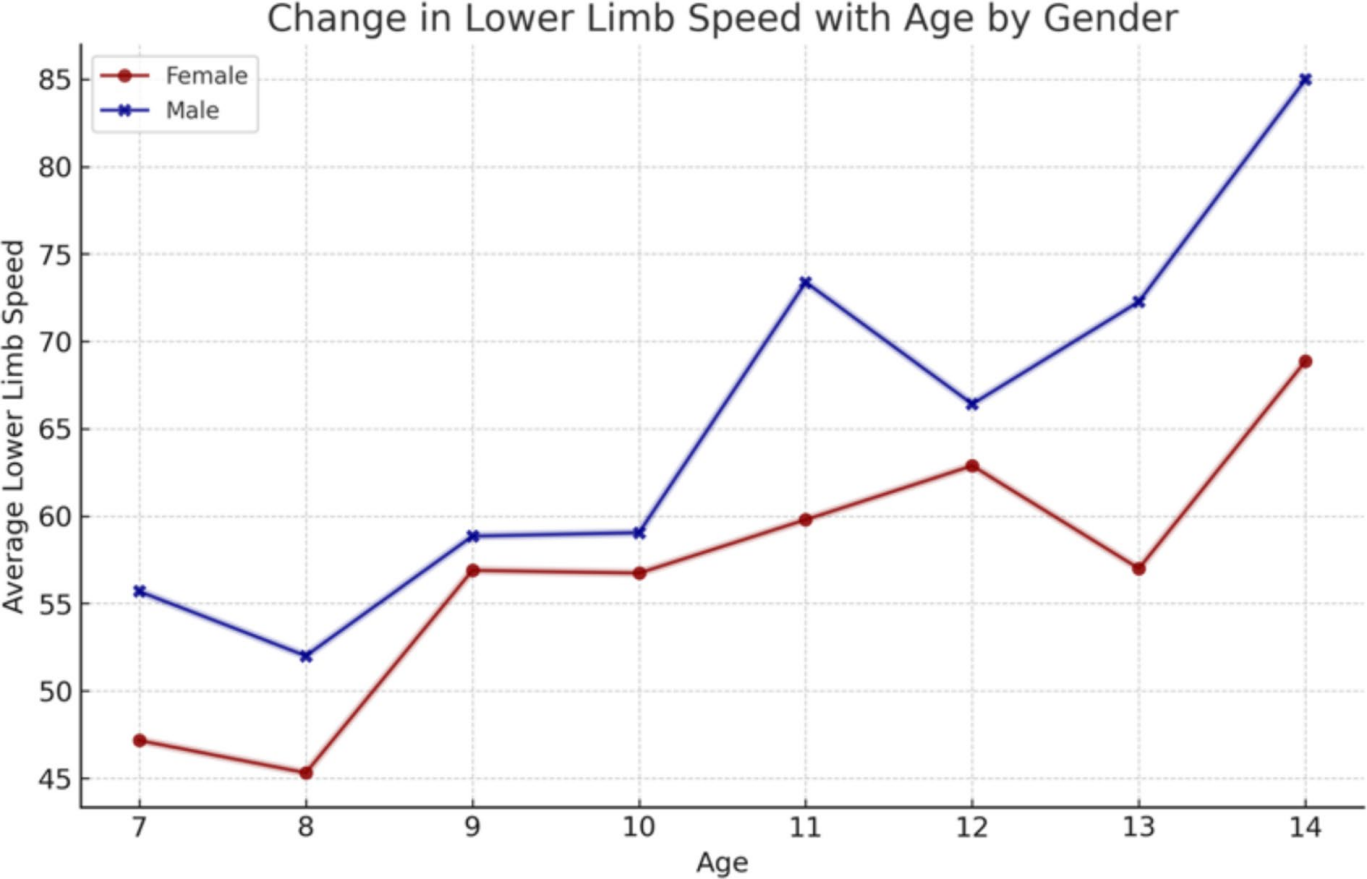
Impact of age on lower limb velocity, with a particular focus on the influence of sex.

The assessment of lower limb speed revealed a statistically significant improvement in performance with age for both boys and girls (Figure 5).

**Figure 6:**
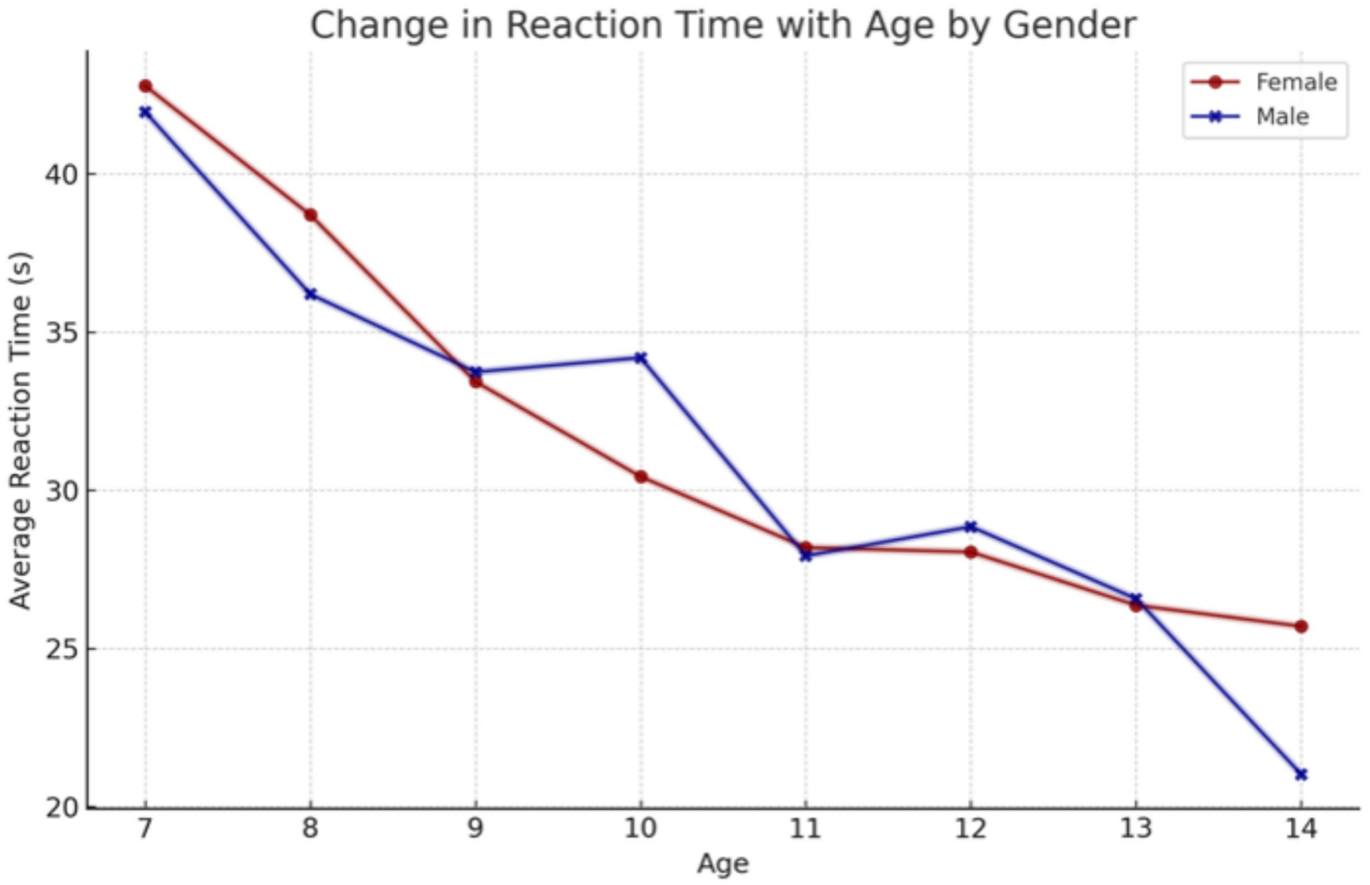
Influence of age on response time, with a particular focus on sex differences.

Similar to the results for hand-grip strength are the results for reaction time. The older the children, the significantly shorter the reaction time to the stimulus (Figure 6).

**Figure 7:**
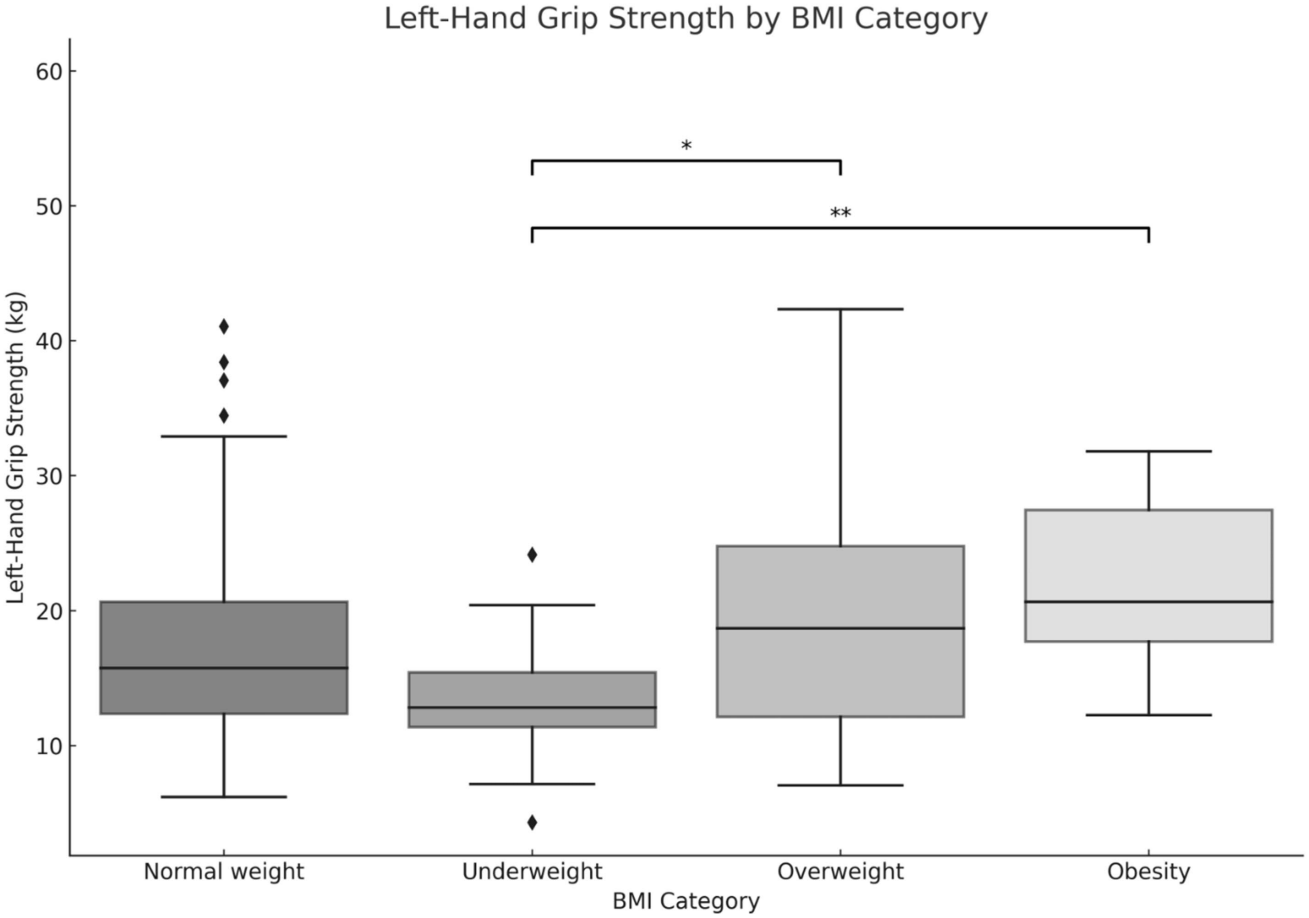
Relationship between body mass index (BMI) and left limb hand-grip strength. *p < 0.05, **p < 0.01, ***p < 0.001.

The subsequent phase of the study was to ascertain whether there is a correlation between BMI and the physical fitness of the children under examination. No statistically significant differences were observed between the groups in terms of balance and reaction time (*p* > 0.05). A significant difference was observed in all other indices of physical fitness (*p* < 0.05). Subjects with a BMI indicative of overweight status demonstrated the most favorable outcomes in both the right (23.7 kg) and left (21.9 kg) hand grip strength assessments. Those who were overweight exhibited gains of 20 kg for the right hand and 19.5 kg for the left hand, respectively. Children with a normal body weight demonstrated a mean gain of 18.4 kg for the right hand and 17.3 kg for the left hand. The lowest values were observed in subjects with underweight status, with a mean of 13.5 kg for the right hand and 13.4 kg for the left hand. The differences between the groups were statistically significant for both right and left limb results (*p* < 0.01). A comparison of the squeeze strength of the left limb revealed significant differences between underweight and overweight and obese children (Figure 7).

**Figure 8:**
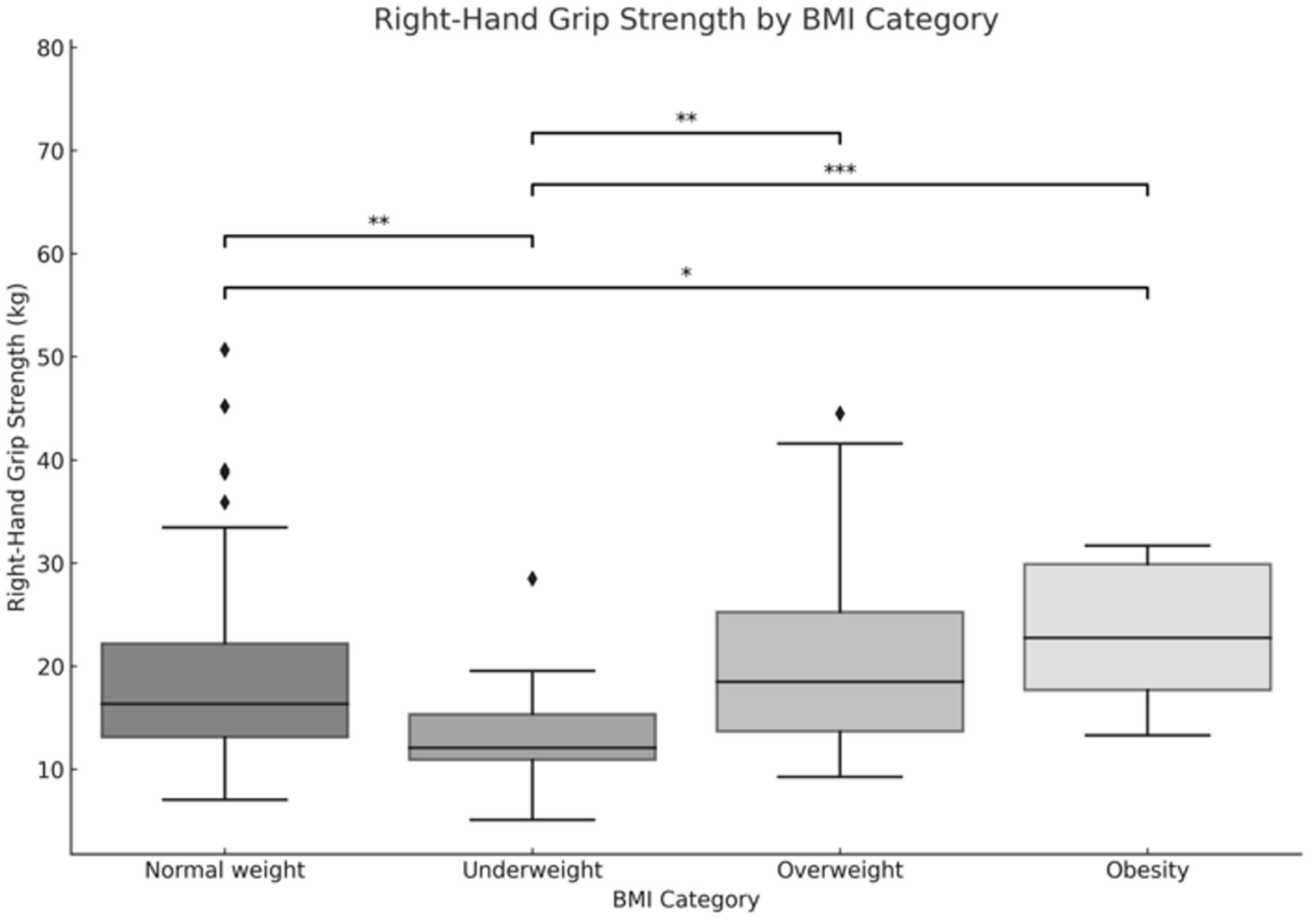
Relationship between body mass index (BMI) and right limb hand-grip strength. *p < 0.05, **p < 0.01, ***p < 0.001.

The results for right limb grip strength demonstrate the most pronounced disparity between underweight and obese children (*p* < 0.001). Additionally, a notable distinction was observed between underweight and overweight children (*p* < 0.01). Children with a normal weight exhibited a significantly stronger grip than underweight children (*p* < 0.01), yet a significantly weaker grip than obese children (*p* < 0.05) (Figure 8).

**Figure 9:**
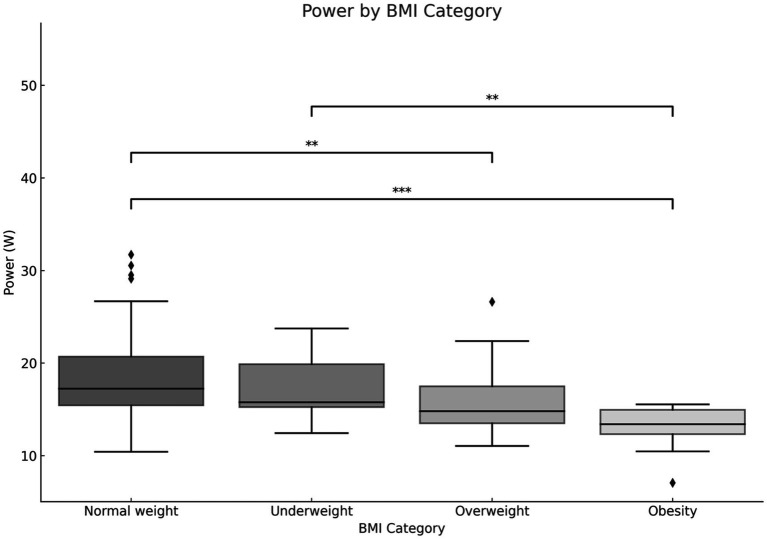
Maximum power output is dependent on the individual’s body mass index (BMI). *p < 0.05, **p < 0.01, ***p < 0.001.

The results of the maximum power analysis indicated that children with a normal body weight exhibited the most favorable outcomes. The results presented for maximal power demonstrate that any BMI disorder is associated with a diminished capacity to develop maximal power (Figure 9). The most pronounced differences were observed between the group with normal BMI and the group with obesity (*p* < 0.001). Additionally, a notable distinction was evident between the normal and obese BMI groups and the underweight and obese group (*p* < 0.01) (Figure 9).

The next step was to compare the results from both testing sessions. The outcomes from both test occasions and the discrepancies that emerged are presented in the following table (Table 5). It was evident that notable enhancements were evident in both right and left limb hand-grip strength, reaction time and lower limb speed (*p* < 0.001). Conversely, no considerable discrepancies were observed in the results for maximal power and balance (*p* > 0.05).

**Table 5:** A comparative analysis of the results obtained from the fitness tests conducted at the initial and subsequent examination periods.

Test	Stage 1	Stage 2	Difference	*P* – value	*d*-Cohen
Right-hand strength (kg)	17.73	19.22	1.49	<0.001	−0.566
Left-hand strength (kg)	17.05	18.15	1.10	<0.001	−0.416
Reaction time (s)	36.36	29.79	−6.56	<0.001	0.942
Leg speed (repetitions)	55.11	63.01	7.90	<0.001	−0.567
Balance (ppm)	733.30	827.96	94.65	0.310	0.112
Leg power (W)	17.51	16.89	−0.63	0.751	−0.112

Subsequently, an investigation was conducted to ascertain whether children who engage in extracurricular sports would exhibit enhanced performance in physical fitness assessments (Table 6). Children who participated in extracurricular sporting activities exhibited superior performance in all assessed domains, including hand-grip strength (right and left), reaction time, and lower limb speed (*p* < 0.05). No statistically significant differences were observed in the results of the balance and lower limb maximal power tests.

**Table 6:** A comparative analysis of the results of fitness tests conducted on children with varying levels of physical activity.

Test	Active	Non-active	Difference	*P*-value	Effect size (*r*)
Right-hand strength (kg)	19.33	16.74	2.59	0.020	0.1756
Left-hand strength (kg)	18.04	16.44	1.60	0.096	0.1270
Reaction time (s)	33.99	37.89	−3.84	0.015	0.2339
Leg speed (repetitions)	61.62	51.07	10.55	<0.001	0.4141
Balance (ppm)	660.30	778.64	−118.34	0.223	0.1254
Leg power (W)	17.64	17.44	0.20	0.494	0.0523

The final stage of the analysis was to ascertain whether a relationship existed between the variables. Statistically significant correlations were identified between the hand-grip strength of both limbs and the other variables (Table 7).

**Table 7:** The objective of this study is to evaluate the relationship between the variables under investigation.

Variable 1	Variable 2	*r*-Spearman	*p*-value
Right-hand strength (kg)	Left-hand strength (kg)	0.91	<0.001
Right-hand strength (kg)	Reaction time (s)	−0.63	<0.001
Right-hand strength (kg)	Balance (ppm)	−0.50	<0.001
Right-hand strength (kg)	Leg power (W)	−0.22	0.022
Right-hand strength (kg)	Leg speed (repetitions)	0.40	<0.001
Left-hand strength (kg)	Reaction time (s)	−0.61	<0.001
Left-hand strength (kg)	Balance (ppm)	−0.53	<0.001
Left-hand strength (kg)	Leg power (W)	−0.12	0.082
Left-hand strength (kg)	Leg speed (repetitions)	0.43	<0.001
Reaction time (s)	Balance (ppm)	0.41	<0.001
Reaction time (s)	Leg power (W)	0.10	0.048
Reaction time (s)	Leg speed (repetitions)	−0.30	<0.001
Balance (ppm)	Leg power (W)	0.10	0.041
Balance (ppm)	Leg speed (repetitions)	−0.42	<0.001
Leg power (W)	Leg speed (repetitions)	0.00	0.569

## 4 Discussion

The findings of our study elucidate the process of physical fitness development in school-aged children. The data set includes age-related changes for both boys and girls, which allows for the differentiation of the rate of change between the sexes. It was observed that the development of upper limb grip strength occurs at a similar rate in both sexes up to the age of 12, after which point a further dynamic increase is evident in boys and a period of stagnation is observed in girls. The development of lower limb speed occurs at a comparable pace in both sexes. However, from the outset of their schooling, boys demonstrate superior performance, which is sustained over time. The assessment of lower limb power yielded disparate results, yet a discernible pattern emerged across both sexes. The capacity to maintain static balance exhibited a progressive enhancement with each additional year of age in both boys and girls. However, at the age of 10, a discernible divergence emerged, with girls demonstrating superior performance compared to boys. These findings are consistent with research conducted by Cortés-Rojas et al. (31), who observed that girls aged 7–12 performed better in balance and reaction time tasks, while boys exhibited superior grip strength and lower body explosive power. Similarly, Mavingire et al. (32) reported gender differences in balance and strength among 10–12-year-old children, with BMI playing a moderating role. The analysis of the physical fitness results in conjunction with BMI revealed that children with higher body weights demonstrated superior performance in the hand-grip strength test of both the right and left limbs. Conversely, when maximum power was evaluated, children with a normal BMI demonstrated the most optimal results. No differences in performance were observed in lower limb speed, reaction time or balance between children with different BMI values. Comparable findings were reported by Hermassi et al. (33), who found that while children with higher BMI performed better in grip strength, those with normal BMI had better outcomes in flexibility, sprinting and jumping ability. Sui et al. (62) also noted significant deficits in coordination and balance among overweight children aged 9–10, emphasizing the negative impact of excess body weight on motor control. These outcomes are consistent with the findings of Hsu et al. (34), who showed that BMI z-score, body fat percentage, and muscle weight significantly affect performance in various physical fitness tests (800-m run, sit-ups, standing long jump) in 10-year-old children, reinforcing the importance of body composition in fitness profiling. In examining the interrelationships between individual physical fitness scores, particular attention was paid to upper limb grip strength, which exhibited correlations with nearly every other test. The children’s performance in the upper limb squeeze strength test was found to be a significant predictor of their performance in the other physical fitness tests. Reaction time scores demonstrated correlations with balance, power, and lower limb speed scores, though these correlations were weak to moderate. Laul et al. (35) also highlighted a significant correlation between grip strength and reaction time as well as balance in children aged 9–13. Moreover, Nieczuja-Dwojacka et al. (36) demonstrated that handgrip strength in prepubertal children is strongly associated with muscle endurance and simple reaction time, reinforcing its value as a general fitness indicator. A study by Fraser et al. in which subjects underwent dynamometric measurements (including hand squeeze strength) on multiple occasions over a 20-year period demonstrated that strength in childhood was significantly correlated with strength in adulthood (37). It can therefore be posited that the strength demonstrated by a child in a hand-grip strength test may serve as an indicator of their strength in adulthood. Abe et al. observed the development of strength in pre-school children and, in a similar manner to our own study, demonstrated an increase in strength (exceeding 3 kg of squeeze strength) in both male and female subjects. Furthermore, the Abe study indicated that, at the pre-school age, boys exhibited greater strength than girls (38). In contrast, Gómez-Campos et al. (39) reached a different conclusion. In their study, they reported grip strength results for subjects aged 6–80 years. No differences in grip strength were observed in children aged 6–11 years old based on gender. The discrepancy commenced after the age of 12 years, with males exhibiting greater strength than females (39). These longitudinal and cross-sectional trends support the findings of Hermassi et al. (33), who confirmed a gradual increase in grip strength among boys aged 11–13 and emphasized its correlation with agility and academic indicators. Moreover, Cortés-Rojas et al. (31) provided additional evidence that the emergence of sex-related differences in strength becomes more pronounced after age 11. Matsudo et al. (40) demonstrated analogous correlations between strength and other fitness assessments. The researchers indicated that grip strength in children aged 10–17 years was associated with superior outcomes in tests of speed, agility, high jump, and flexibility (40). The study by Reigal et al. (41) examined the relationship between simple and complex reaction time and physical activity. The findings indicated that performance on reaction time tests was associated with performance on other fitness tests and physical activity levels (41). Zhang et al. (42) demonstrated that incorporating additional physical activity can effectively enhance reaction time in children. Similarly, Laul et al. (35) found that balance and reaction time are interrelated and influenced by BMI and sex. A comparison of the results of physical fitness tests by BMI revealed significant differences. Tsolakis et al. (43) present similar findings in their study, demonstrating significant differences between children with normal BMI and those who are obese in physical fitness tests. Furthermore, a positive correlation was observed between BMI and hand squeeze strength, indicating that children with higher body weight exhibited greater hand squeeze strength. In other tests, such as the long jump, shuttle run and abdominal muscular endurance, children with a normal body weight demonstrated superior performance (43). In their study, Chen et al. examined the relationship between BMI and the results of physical tests, including grip strength, long jump, sit and reach, sprinting, and endurance running. They found that children with a normal BMI exhibited superior performance in these tests. Grip strength was the exception to this rule, with children with a higher BMI performing better than those with a normal BMI. All of the results described by Chen improved with the age of the children tested (44). Higher BMI was also associated with poorer performance in the long jump and 30-meter sprint tests, as observed by Dinc et al. (45). In line with this, findings by Mavingire et al. (32) and Sui et al. (62) both emphasize that children with elevated BMI perform worse in balance and coordination-based tasks, despite showing greater grip strength. These results reinforce the multifaceted influence of body composition on different components of physical fitness. Contreras-Osorio et al. (46) further demonstrated that higher BMI and waist-to-height ratios were negatively associated with executive function performance, such as cognitive flexibility and working memory, while muscular fitness (e.g., grip strength and jumping ability) predicted better cognitive scores. Additionally, Li described age and gender differences in static balance among children. His findings indicated that girls exhibited superior static and dynamic balance compared to boys in testing (47). In a study by Ługowska et al. (48), it was demonstrated that elevated levels of physical activity in children were linked to enhanced test outcomes for strength, running speed and jumping distance. The study by Cieśla et al. (49) sought to ascertain whether spontaneous and additional structured physical activity affect children’s physical fitness. The results demonstrated a positive correlation between spontaneous physical activity and the outcomes of tests of flexibility, explosive leg strength and arm strength. Furthermore, additional organized physical activity was found to be positively related to the results of trunk muscle strength and explosive leg strength. It was observed that there were significant differences in the performance of the two sexes on various tests of physical strength and flexibility. Furthermore, it was observed that male participants exhibited superior performance in tests of trunk muscle strength, explosive leg strength, and arm strength, whereas female participants demonstrated enhanced flexibility. The results of all fitness tests were adversely affected by BMI, irrespective of the age of the subjects (49). These observations are supported by Korcz et al. (50), who showed that children aged 8–9 years with higher levels of structured and spontaneous physical activity achieved better results in school-related physical tasks, particularly those involving coordination, flexibility and strength. Furthermore, Cortés-Rojas et al. (31) confirmed that female children generally outperform males in balance and executive function, which correlates with physical control measures. A study by Baj-Korpak et al. (51) evaluated the impact of supplementary physical activity on physical fitness, comprising shuttle running, long jump, medicine ball throw and 4-min run. The study group that participated in additional physical activity achieved superior results in all tests when compared to the control group that did not engage in additional physical activity (51). Fang et al. (52) conducted a study to analyze the physical activity of preschool children and concluded that the level of physical activity is an effective predictor of children’s physical fitness. The higher the physical activity level of the children studied, the more favorable the results they obtained in physical fitness tests (52). Many therapies for obesity are currently being explored (53), but increased physical activity appears to be one of the most promising. However, it should also be noted, as emphasized by Lupo et al. (54), that the most physically active children are not always the fittest. Their study found only weak-to-moderate correlations between physical activity levels (PAQ-C) and test outcomes such as grip strength, sprinting and flexibility, suggesting that activity alone may not guarantee fitness across all domains. This study has several limitations. A high proportion of children (62%) did not participate in extracurricular physical activities, which may have influenced their physical fitness levels and affected the overall interpretation of the results. In addition, external factors such as diet, socioeconomic background, and screen time were not controlled. These variables can significantly impact physical development and may have acted as confounding factors. Finally, the relatively short study duration (4 months) may not have been sufficient to capture more distinct or long-term changes in physical fitness. Future studies should consider longer observation periods and include lifestyle-related variables to improve the robustness of the findings.

## 5 Conclusion

The aim of this study was to compare the results of physical fitness tests in relation to gender, age, BMI, and participation in extracurricular sports activities. The hypothesis that children involved in extracurricular physical activity would achieve better fitness outcomes was partially confirmed. Similarly, the assumption that abnormal BMI would be associated with poorer physical performance was also partially supported. In contrast, the hypothesis that higher performance in one fitness component would correlate with better outcomes in other tests was confirmed. As expected, the four-month school period was associated with improvements in most of the assessed motor abilities, indicating a positive impact of regular physical education and growth-related development.

## Data Availability

The datasets presented in this study can be found in online repositories. The names of the repository/repositories and accession number(s) can be found: https://doi.org/10.18150/9AQZKJ.

## References

[1] Fonseca APLM Azevedo CVMD Santos RMR. Sleep and health-related physical fitness in children and adolescents: a systematic review. Sleep Sci. (2021) 14:357–65. doi: 10.5935/1984-0063.20200125, PMID: 35087633 PMC8776269

[2] Kubusiak-Słonina A Grzegorczyk J Mazur A. Ocena sprawności i aktywności fizycznej dzieci szkolnych z nadmierną i prawidłową masą ciała Endokrynol. Otył Zab Przem Mat. (2012) 8:16–23.

[3] Puchalska-Sarna A Baran R Kustra M Pop T Herbert J Baran J. The level and factors differentiating the physical fitness of adolescents passively and actively resting in south-eastern Poland—a pilot study. Children. (2022) 9:1341. doi: 10.3390/children9091341, PMID: 36138650 PMC9497588

[4] American College of Sports Medicine Liguori G Feito Y Fountaine C Roy B. ACSM’s guidelines for exercise testing and prescription. Philadelphia: Wolters Kluwer (2021).

[5] Szymala M. Aktywność fizyczna i jej wpływ na rozwój dzieci i młodzieży w wybranych badaniach ankietowych. Aktywność Ruchowa Ludzi w Różnym Wieku. (2021) 1-4:13–23.

[6] James J Pringle A Mourton S Roscoe CMP. The effects of physical activity on academic performance in school-aged children: a systematic review. Children. (2023) 10:1019. doi: 10.3390/children10061019, PMID: 37371251 PMC10297707

[7] Aniśko B Siatkowski I Wójcik M. Body mass composition analysis as a predictor of overweight and obesity in children and adolescents. Front Public Health. (2024) 12:1371420. doi: 10.3389/fpubh.2024.1371420, PMID: 38721538 PMC11076875

[8] Tan VPS Macdonald HM Kim SJ Nettlefold L Gabel L Ashe MC. Heather a McKay, influence of physical activity on bone strength in children and adolescents: a systematic review and narrative synthesis. J Bone Miner Res. (2014) 29:2161–81. doi: 10.1002/jbmr.2254, PMID: 24737388

[9] Soler-Lanagrán A Castañeda-Vázquez C. Sedentary lifestyle and health risks in children. A systematic review. J Sport Health Res. (2017) 9:187–98.

[10] Laurent CW Burkart S Andre C RMC S. Physical activity, fitness, school readiness, and cognition in early childhood: a systematic review. J Phys Act Health. (2021) 18:1004–13. doi: 10.1123/jpah.2020-0844, PMID: 34140418 PMC9297301

[11] Li B Ng K Tong X Zhou X Ye J Yu JJ. Physical activity and mental health in children and youth during COVID-19: a systematic review and meta-analysis. Child Adolesc Psychiatry Ment Health. (2023) 17:92. doi: 10.1186/s13034-023-00629-4, PMID: 37468975 PMC10357657

[12] Chaput J-P Willumsen J Bull F Chou R Ekelund U Firth J. 2020 WHO guidelines on physical activity and sedentary behaviour for children and adolescents aged 5–17 years: summary of the evidence. Int J Behav Nutr Phys Act. (2020) 17:141. doi: 10.1186/s12966-020-01037-z, PMID: 33239009 PMC7691077

[13] Mitchell JA. Physical inactivity in childhood from preschool to adolescence. ACSMs Health Fit J. (2019) 23:21–5. doi: 10.1249/FIT.0000000000000507, PMID: 32863707 PMC7451199

[14] WHO guidelines on physical activity and sedentary behaviour. (2020). Geneva: World Health Organization. 33369898

[15] Huang S-F Duan H-Y. Study on sports, extracurricular activities, electronic device usage factors associated with chronic fatigue syndrome in Taiwanese preschoolers. Children. (2023) 10:1278. doi: 10.3390/children10081278, PMID: 37628277 PMC10453614

[16] Dunleavy K Kubo SA. Therapeutic exercise prescription. Philadelphia, PA: Elsevier (2019).

[17] Krzywicka-Michałowska M Dylewicz P Wilk M. Metody oceny siły i wytrzymałości mięśniowej w kontekście doboru intensywności i oceny efektywności treningu oporowego w rehabilitacji kardiologicznej. Kardiologia Polska, 68:695–700. 22006623

[18] McGinnis PM Winiarski S Buśko K Gajewski J Michnik R Rutkowska-Kucharska A. Biomechanika w sporcie i ćwiczeniach ruchowych. Wrocław: Edra Urban & Partner (2021).

[19] Latorre-Román PÁ Mora-López D García-Pinillos F. Intellectual maturity and physical fitness in preschool children. Pediatr Int. (2016) 58:450–5. doi: 10.1111/ped.12898, PMID: 26714789

[20] Guthold R Stevens GA Riley LM Bull FC. Global trends in insufficient physical activity among adolescents: a pooled analysis of 298 population-based surveys with 1·6 million participants. Lancet Child Adoles Health. (2020) 4:23–35. doi: 10.1016/S2352-4642(19)30323-2, PMID: 31761562 PMC6919336

[21] Kann L McManus T Harris WA Shanklin SL Flint KH Queen B. Youth risk behavior surveillance — United States, 2017. MMWR Surveill Summ. (2018) 67:1–114. doi: 10.15585/mmwr.ss6708a1, PMID: 29902162 PMC6002027

[22] Kellstedt DK Schenkelberg MA Essay AM Von Seggern MJ Rosenkranz RR Welk GJ. Youth sport participation and physical activity in rural communities. Arch Public Health. (2021) 79:46. doi: 10.1186/s13690-021-00570-y, PMID: 33832548 PMC8028731

[23] Howells K Coppinger T. Children’s perceived and actual physical activity levels within the elementary school setting. Int J Environ Res Public Health. (2021) 18:3485. doi: 10.3390/ijerph18073485, PMID: 33801656 PMC8037387

[24] Masanovic B Gardasevic J Marques A Peralta M Demetriou Y Sturm DJ. Trends in physical fitness among school-aged children and adolescents: a systematic review. Front Pediatr. (2020) 8:627529. doi: 10.3389/fped.2020.627529, PMID: 33363072 PMC7759499

[25] Nałęcz H Mazur J Fijałkowska A. Niedostateczny poziom aktywności fizycznej w Polsce jako zagrożenie i wyzwanie dla zdrowia publicznego. In: Drygas W Gajewska M Zdrojewski T, editors. Raport Komitetu Zdrowia Publicznego PAN. Warszawa: Narodowy Instytut Zdrowia Publicznego–Państwowy Zakład Higieny. (2021) p. 69–90.

[26] Active Living Research. Attendance in physical education classes, sedentary behavior, and different forms of physical activity among schoolchildren: a cross-sectional study. Active living research. (2015). Available online at: https://activelivingresearch.org/sites/default/files/ALR_Brief_ActiveEducation_Jan2015.pdf (accessed July 21, 2024) 10.1186/s12889-022-13864-9 PMC9341117 35915433

[27] De Jesus GM De Oliveira Araujo RH Dias LA Barros AKC Dos Santos Araujo LDM De Assis MAA. Attendance in physical education classes, sedentary behavior, and different forms of physical activity among schoolchildren: a cross-sectional study. BMC Public Health. (2022) 22:1461. doi: 10.1186/s12889-022-13864-9, PMID: 35915433 PMC9341117

[28] Madejski E Kosiba G Madejski R. Uczniowskie opinie o lekcji wychowania fizycznego In: Polechoński J Skalik K, editors. Współczesne problemy wychowania fizycznego. Katowice: Akademia Wychowania Fizycznego im. Jerzego Kukuczki. (2021). 71.

[29] Woynarowska B Mazur J Oblacińska A. (2015). Uczestnictwo uczniów w lekcjach wychowania fizycznego w szkołach w Polsce. Hygeia Public Health, (2015) 50:183–90.

[30] Wickham H Grolemund G. R for data science: import, tidy, transform, visualize, and model data. Sebastopol, CA: O’Reilly (2016).

[31] Cortés-Rojas R Castellano-Ruiz MI Baeza-Medina A Gil-Espinosa FJ Álvarez-Salvago F Jiménez-García JD. Associations of physical fitness with cognitive performance in children aged 7–12 years: a cross-sectional study. Appl Sci. (2024) 14:4965. doi: 10.3390/app14124965

[32] Mavingire CC Hans De Ridder J Makaza D Monyeki MA. Health-related physical fitness, anthropometry and physical activity levels of Zimbabwean children aged 10–12 years old. Master's Thesis North-West University. (2018) 28:299–317. doi: 10.37597/ajphes.2022.28.4.2

[33] Hermassi S Ketelhut S Konukman F Sellami M Al-Marri S Nigg CR. Comparative analysis of physical activity, performance-related health, and academic achievements in 11-to-13-year-old schoolchildren in Qatar. Healthcare. (2024) 12:588. doi: 10.3390/healthcare12050588, PMID: 38470699 PMC10930824

[34] Hsu CY Chen LS Chang IJ Fang WC Huang SW Lin RH. Can anthropometry and body composition explain physical fitness levels in school-aged children? Children. (2021) 8:460. doi: 10.3390/children8060460, PMID: 34072785 PMC8229107

[35] Laul T Sharma S Kumar A. Correlation between dynamic balance and reaction time in children between age 9–13 years: an observational study. Int J Health Sci Res. (2021) 10:166–70. doi: 10.21275/SR211002160344

[36] Nieczuja-Dwojacka J Marchewka J Siniarska A Budnik A Popielarz K Tabak I. Influence of body build on hand grip strength, simple reaction time and strength of the abdominal muscles in prepubertal children. Anthropol Anz. (2023) 80:151–8. doi: 10.1127/anthranz/2023/1591, PMID: 36752666

[37] Fraser BJ Blizzard L Buscot M-J Schmidt MD Dwyer T Venn AJ. Muscular strength across the life course: the tracking and trajectory patterns of muscular strength between childhood and mid-adulthood in an Australian cohort. J Sci Med Sport. (2021) 24:696–701. doi: 10.1016/j.jsams.2021.01.011, PMID: 33640263

[38] Abe A Sanui R Loenneke JP Abe T. One-year handgrip strength change in kindergarteners depends upon physical activity status. Life. (2023) 13:1665. doi: 10.3390/life13081665, PMID: 37629522 PMC10455176

[39] Gómez-Campos R Vidal Espinoza R De Arruda M Ronque ERV Urra-Albornoz C Minango JC. Relationship between age and handgrip strength: proposal of reference values from infancy to senescence. Front Public Health. (2023) 10:1072684. doi: 10.3389/fpubh.2022.1072684, PMID: 36777772 PMC9909206

[40] Matsudo VKR Matsudo SM Rezende LFMD Raso V. Força de preensão manual como preditor de aptidão física em crianças e adolescentes. Revista Brasileira de Cineantropometria e Desempenho Humano. (2014) 17:1. doi: 10.5007/1980-0037.2015v17n1p1

[41] Reigal RE Barrero S Martín I Morales-Sánchez V Juárez-Ruiz De Mier R Hernández-Mendo A. Relationships between reaction time, selective attention, physical activity, and physical fitness in children. Front Psychol. (2019) 10:2278. doi: 10.3389/fpsyg.2019.02278, PMID: 31681086 PMC6803537

[42] Zhang W Wang X Li X Yan H Song Y Li X. Effects of acute moderate-intensity aerobic exercise on cognitive function in E-athletes: a randomized controlled trial. Medicine. (2023) 102:e35108. doi: 10.1097/MD.0000000000035108, PMID: 37800783 PMC10553036

[43] Tsolakis C Cherouveim ED Skouras AZ Antonakis-Karamintzas D Czvekus C Halvatsiotis P. The impact of obesity on the fitness performance of school-aged children living in rural areas—the West Attica project. Int J Environ Res Public Health. (2022) 19:11476. doi: 10.3390/ijerph191811476, PMID: 36141749 PMC9517351

[44] Chen G Chen J Liu J Hu Y Liu Y. Relationship between body mass index and physical fitness of children and adolescents in Xinjiang, China: a cross-sectional study. BMC Public Health. (2022) 22:1680. doi: 10.1186/s12889-022-14089-6, PMID: 36064657 PMC9442906

[45] Dinç N Güzel P Özbey S Beşikçi T Seyhan S Kalkan N. Obesity prevalence and physical fitness in school-aged children. Univ J Educ Res. (2019) 7:659–63. doi: 10.13189/ujer.2019.070303

[46] Contreras-Osorio F Guzmán-Guzmán IP Cerda-Vega E Chirosa-Ríos L Ramírez-Campillo R Campos-Jara C. Anthropometric parameters, physical activity, physical fitness, and executive functions among primary school children. Int J Environ Res Public Health. (2022) 19:3045. doi: 10.3390/ijerph19053045, PMID: 35270736 PMC8910200

[47] Li R Liu M Zhu J Li R Zhao H Zhang L. Age and gender differences in static and dynamic balance of Chinese preschool children. Front Physiol. (2022) 13:1013171. doi: 10.3389/fphys.2022.1013171, PMID: 36324303 PMC9618940

[48] Ługowska K Kolanowski W Trafialek J. Increasing physical activity at school improves physical fitness of early adolescents. Int J Environ Res Public Health. (2023) 20:2348. doi: 10.3390/ijerph20032348, PMID: 36767711 PMC9915395

[49] Cieśla E Mleczko E Bergier J Markowska M Nowak-Starz G. Health-related physical fitness, BMI, physical activity and time spent at a computer screen in 6 and 7-year-old children from rural areas in Poland. Ann Agric Environ Med. (2014) 21:617–21. doi: 10.5604/12321966.1120613, PMID: 25292140

[50] Korcz A Bojkowski Ł Bronikowski M Łopatka M Khorkova M Koszałka-Silska A. Cluster analysis of physical activity and physical fitness and their associations with components of school skills in children aged 8-9 years. Sci Rep. (2025) 15:5053. doi: 10.1038/s41598-025-88359-9, PMID: 39934310 PMC11814301

[51] Baj-Korpak J Zaworski K Stelmach MJ Lichograj P Wochna M. Sports activity and changes in physical fitness of polish children and adolescents: OSF study. Front Pediatr. (2022) 10:976943. doi: 10.3389/fped.2022.976943, PMID: 36452358 PMC9703092

[52] Fang H Quan M Zhou T Sun S Zhang J Zhang H. Relationship between physical activity and physical fitness in preschool children: a cross-sectional study. Biomed Res Int. (2017) 2017:1–8. doi: 10.1155/2017/9314026, PMID: 29359160 PMC5735582

[53] Placek K Aniśko B Wójcik M. Application of balneological treatment in the treatment of obesity in children. Acta Balneol. (2023) 65:340–3. doi: 10.36740/ABaL202305114

[54] Lupo C De Pasquale P Boccia G Ungureanu AN Moisè P Mulasso A. The most active child is not always the fittest: physical activity and fitness are weakly correlated. Sports. (2023) 11:3. doi: 10.3390/sports11010003, PMID: 36668707 PMC9866618

[55] Heyward VH. Advanced Fitness Assessment and Exercise Prescription (3rd ed.). Champaign, IL: Human Kinetics. (1998).

[56] Chang SP Chen YH. Relationships between sleep quality, physical fitness and body mass index in college freshmen. J Sports Med Phys Fitness. (2015) 55:1234–41. PMID: 25323481

[57] Committee on Physical Activity and Physical Education in the School Environment. Educating the student body: taking physical activity and physical education to school. (2013), Washington D.C.: National Academies Press. 24851299

[58] Felten DL O’Banion MK Maida MS Netter FH Perkins JA CAG M. Netter atlas neuroanatomii i neurofizjologii. Wrocław: Edra Urban & Partner (2023).

[59] Polechoński J Skalik K, Akademia Wychowania Fizycznego im. Jerzego Kukuczki (Katowice) eds. Współczesne problemy wychowania fizycznego. Cz. 4. Katowice: Wydawnictwo Akademii Wychowania Fizycznego (2021).

[60] Lankau A Krajewska-Kułak E. Sytuacje trudne w ochronie zdrowia. Tom VI. Białystok: Uniwersytet Medyczny w Białymstoku (2021).

[61] World Health Organization. Global recommendations on physical activity for health. Geneva: WHO Press. (2010). 58 p. 26180873

[62] Sui Y Cui L Jia B Ding X He M Da Y. Correlation between gross motor coordination and basic coordination capacities in normal-weight and overweight/obese children aged 9–10 years. PeerJ (2024) 12:e17865. doi: 10.7717/peerj.17865 39135953 PMC11318586

